# Cytokinin availability and chloroplast functional lifetime in senescing leaves

**DOI:** 10.3389/fpls.2026.1901011

**Published:** 2026-07-27

**Authors:** Veronika Kábrtová, Zuzana Kučerová, Beata Budíková, Martin Hudeček, Martina Špundová, Ondřej Plíhal

**Affiliations:** 1 Laboratory of Growth Regulators, Faculty of Science, Palacký University Olomouc, Olomouc, Czechia; 2 Laboratory of Growth Regulators, Institute of Experimental Botany of the Czech Academy of Sciences, Olomouc, Czechia; 3 Department of Biophysics, Faculty of Science, Palacký University Olomouc, Olomouc, Czechia

**Keywords:** *Arabidopsis thaliana*, chloroplast dismantling, cytokinin transport, cytokinins, leaf senescence, nitrogen remobilization, photosynthetic maintenance, reactive oxygen species

## Abstract

Leaf senescence is often characterized by visible yellowing, reflecting the degradation of photosynthetic pigments and the functional deterioration of photosynthetic complexes, marking a shift from photosynthetic maintenance to nutrient remobilization. Cytokinins are key modulators in this process. This review examines cytokinin action in mature Arabidopsis leaves from the perspective of chloroplast functional lifetime, focusing on how long-distance supply, local metabolism and candidate transport routes may shape cytokinin availability and action during leaf senescence. Cytokinin signaling is further considered in relation to nitrogen status, because chloroplasts are both sites of photosynthetic carbon assimilation and major reservoirs of remobilizable nitrogen. As senescence progresses, chloroplasts are not merely passive targets of degradation. Declining photosynthetic function can generate retrograde signals, alter reactive oxygen species homeostasis, and promote transcriptional programs that accelerate dismantling of the photosynthetic apparatus. Within this framework, cytokinins help preserve chlorophyll content, photosynthetic complexes, Photosystem II function, and chloroplast-related gene expression while buffering redox- and light-dependent feedbacks. Cytokinin action in mature leaves is therefore discussed here mainly in the context of spatiotemporal regulation of chloroplast functional lifetime. Cytokinin availability, nitrogen status, and light-dependent feedback from the photosynthetic apparatus jointly influence when photosynthetic maintenance gives way to chloroplast dismantling and nutrient remobilization. This perspective also reconciles recent evidence from detached Arabidopsis leaves that cytokinins can transiently repress PSII photochemistry under prolonged darkness while delaying subsequent chlorophyll loss, adding another layer to cytokinin-mediated protection during early stages of senescence.

## 1 Introduction

Mature leaves must remain productive long enough to support growth, yet eventually their nitrogen (N)-rich chloroplast proteins become valuable resources for developing tissues (Diaz et al., 2008; Fan et al., 2009; Guo et al., 2021; Sakuraba, 2022). Recent chloroplast-centered views of senescence emphasize that this transition is not simply the loss of photosynthetic tissue, but the controlled dismantling of an organelle that supports carbon assimilation, stores N, and generates stress and retrograde signals during functional decline (Mayta et al., 2019; Domínguez and Cejudo, 2021). This makes chloroplasts a natural point of convergence between photosynthetic decline, nutrient recycling, and senescence signaling.

Cytokinins (CKs) act throughout the leaf life cycle, but their dominant function changes with developmental stage. During early shoot and leaf development they support shoot apical meristem activity, leaf primordium formation, and cell proliferation (Werner et al., 2003, 2021; Holst et al., 2011; Skalák et al., 2019). As leaves pass through expansion and approach growth arrest, CIN-TCP transcription factors and the chromatin remodeler BRAHMA promote determinate leaf growth partly by inducing *ARR16*, a type-A response regulator that dampens CK responsiveness (Efroni et al., 2013; Skalák et al., 2019). Once leaf growth is largely completed, CK action shifts toward maintenance in mature leaves, where CKs delay senescence, slow chlorophyll loss, preserve photosynthetic activity, and extend leaf longevity (Kim et al., 2006; Zwack and Rashotte, 2013).

This mature-leaf role is inseparable from chloroplast function. Chloroplasts are among the earliest cellular targets of senescence, undergoing chlorophyll degradation, loss of photosynthetic protein complexes, reduced electron transport capacity, and activation of retrograde stress signaling (Evans et al., 2010; Kuai et al., 2018; Krieger‐Liszkay et al., 2019; Domínguez and Cejudo, 2021; Tanaka and Ito, 2025). They are also the main site of photosynthetic carbon assimilation and a major N store. Consequently, CK-dependent protection of photosynthetic competence must be considered together with the plant’s need to remobilize chloroplast resources.

Here, we place CKs at a regulatory interface between photosynthetic source activity and nutrient salvage. In mature Arabidopsis leaves, systemic CK supply, local activation and redistribution, N status, and chloroplast-derived feedbacks jointly define a functional window during which photosynthetic competence is maintained before the leaf shifts toward chloroplast dismantling and nutrient remobilization. We therefore examine CK action as a regulator of chloroplast functional lifetime, defined here as the period during which ageing leaves retain chloroplast traits relevant to photosynthetic source function before chloroplast dismantling and nutrient remobilization prevail. We consider this lifetime through distinct but connected endpoints, including pigment retention, photosynthetic-complex stability, photochemical competence, carbon-assimilation capacity and the timing of chloroplast-derived N remobilization.

## 2 Local and systemic cytokinin availability in mature leaves

In Arabidopsis, CK supply to mature leaves depends on both systemic and local sources. Root-derived *trans*-zeatin (*t*Z)-type CKs represent a major acropetal long-distance input to the shoot (Hirose et al., 2008; Ko et al., 2014; Zhang et al., 2014), consistent with vascular expression of several isopentenyltransferase (*IPT*) biosynthetic genes in roots (Miyawaki et al., 2004; Takei et al., 2004a, 2004b). However, shoots are not merely passive recipients of root-borne CKs. Several *IPT* genes are also expressed in aerial tissues, and CK-activating Lonely guy (*LOG*) genes are expressed in rosette leaves and immature leaf vasculature, supporting local CK precursor production and activation within the shoot (Miyawaki et al., 2004; Kuroha et al., 2009).

Long-distance transport predominantly supplies recipient tissues with ribosylated CK precursors (Hirose et al., 2008; Nedvěd et al., 2025), whereas local CK output is shaped by membrane transport, LOG-dependent metabolic activation, and cytokinin oxidase/dehydrogenase (CKX)-mediated degradation (Werner et al., 2003; Kuroha et al., 2009). CK ribosides and nucleotides can contribute to mobile or precursor pools, but LOG enzymes convert CK riboside 5’-monophosphates into free-base CKs that can bind and activate AHK receptors (Yamada et al., 2001; Mähönen et al., 2006; Kuroha et al., 2009; Lomin et al., 2015). Conversely, CKX-mediated degradation restricts the duration and amplitude of the signal, as illustrated during senescence by AtNAP-dependent induction of *CKX3* expression (Hu et al., 2021). Thus, transport and metabolism together determine not only how much CK reaches mature leaves, but also where and when bioactive CK pools become available for signaling.

Long-distance CK delivery is partly form-specific, with *t*Z-type CKs, particularly *trans*-zeatin riboside (*t*ZR), enriched in xylem sap, whereas isopentenyladenine (iP)-type pools, including iP and isopentenyladenine riboside (iPR), are more prominent in phloem sap (Hirose et al., 2008). A central component of shootward *t*Z-type CK transport is ATP-binding cassette G14 (ABCG14) (Ko et al., 2014; Zhang et al., 2014; Zhao et al., 2021), although ABCG14 can also contribute to long-distance transport of iP-type CKs (Zhao et al., 2023). Once CKs reach the shoot vasculature, their distribution within leaves is likely refined by additional transport systems. CK ribosides are prominent long-distance transport forms (Hirose et al., 2008) and are unlikely to cross membranes efficiently without carriers (Nedvěd et al., 2025). Members of the Equilibrative Nucleoside Transporter family (ENT) are therefore candidates for CK riboside uptake and local redistribution in leaf tissues. Genetic and transport evidence supports CK riboside transport by ENT3, ENT6, ENT8 and, more recently, ENT1 (Sun et al., 2005; Hirose et al., 2008; Nedvěd et al., 2025; Hudeček et al., 2026). *ENT3*, *ENT6* and *ENT8* are expressed in mature-leaf vasculature, and several *ENT* genes are induced during senescence (Sun et al., 2005; Hirose et al., 2008; Chen et al., 2006; Cornelius et al., 2012). These expression patterns are compatible with possible roles in local CK uptake or redistribution, although their specific contribution to CK availability in senescing leaves remains to be elucidated.

In parallel, Purine Permeases (PUPs) may contribute to the uptake of CK nucleobases, with *PUP2* expression in phloem cells (Bürkle et al., 2003; Cedzich et al., 2008). *ENT1* and *PUP1* expression in hydathodes suggests that these tissues may retrieve or redistribute xylem-delivered CKs at the leaf margin (Bürkle et al., 2003; Bernard et al., 2011; Hudeček et al., 2026), although this has not yet been directly demonstrated. Together with ABCC4, a recently described CK efflux transporter affecting CK-dependent stomatal opening, these examples point to local transport steps that might shape CK distribution within mature leaves; however, further evidence is needed to define their relevance for CK distribution in mature and ageing leaves (Klein et al., 2004; Uragami et al., 2024).

## 3 Nitrogen status and cytokinin availability at the transition from chloroplast maintenance to N remobilization

CK availability in mature leaves is also shaped by nutritional signals. Nitrate acts not only as a N source but also as a signal reporting N availability (Crawford, 1995; Ho et al., 2009). It rapidly induces CK biosynthesis, with IPT3 acting as a key determinant of nitrate-responsive CK production, while CYP735A2 contributes to nitrate-induced accumulation of *t*Z-type CKs (Takei et al., 2004a, 2004b; Maeda et al., 2018). Systemic N signaling further involves ABCG14-dependent root-to-shoot CK translocation (Ko et al., 2014; Zhang et al., 2014; Poitout et al., 2018), while *ABCG14* expression itself responds to nitrate (Abualia et al., 2022). Additional local transport capacity may also be N-responsive, since N deprivation or limitation can induce several *ENT* genes (Li et al., 2003; Zrenner et al., 2009; Cornelius et al., 2012). Together, these findings place nitrate upstream of CK biosynthesis and mobility, because nitrate-responsive *IPT* and *CYP735A* activities can increase shootward *t*Z-type signals, while ABCG14-dependent translocation controls their effective root-to-shoot delivery (Takei et al., 2004a, 2004b; Ko et al., 2014; Zhang et al., 2014; Poitout et al., 2018).

Whereas nitrate-responsive CK biosynthesis and transport mainly reflect external N availability and root-to-shoot communication, CK action during leaf ageing is more closely linked to the timing of photosynthetic maintenance relative to chloroplast dismantling and N remobilization (Jordi et al., 2000; Havé et al., 2017). The photosynthetic competence maintained by CKs depends on an N-rich chloroplast machinery that is also a major target of nutrient remobilization during senescence. A large fraction of leaf N is invested in the photosynthetic apparatus, including light-harvesting chlorophyll-protein complexes, electron transport components, Rubisco and other Calvin-Benson cycle enzymes (Makino and Osmond, 1991; Evans and Clarke, 2019). Under favorable N supply, this investment supports high photosynthetic capacity after leaf expansion (Liu et al., 2018), whereas in mature or senescing leaves, CK action has been reported to delay the decline in chlorophyll content, photosynthetic complexes and photosynthetic capacity (Kim et al., 2006; Talla et al., 2016; Vylíčilová et al., 2016; Kábrtová et al., 2026). Nitrate signaling also intersects with chloroplast maintenance in Arabidopsis. Nitrate-responsive NLP7 activates HB52 and HB54 transcription factors, which in turn promote *VAR2/FtsH2* expression. Because VAR2/FtsH2 contributes to FtsH-dependent turnover of damaged D1/PSII proteins, this pathway helps maintain PSII function and photosynthetic light-energy utilization under high light (Ariga et al., 2022).

When N becomes limiting, the chloroplast-localized investment maintained by CKs also becomes a major source of remobilizable nutrients. Rubisco and thylakoid/photosystem-associated proteins represent major pools of leaf N (Makino and Osmond, 1991; Evans and Clarke, 2019; Havé et al., 2017; Sakuraba, 2022). Therefore, CK-dependent maintenance of chlorophyll and photosynthetic complexes may help preserve aspects of source capacity while an ageing leaf remains photosynthetically useful, but may also postpone the conversion of chloroplast protein reserves into exportable N during senescence (Havé et al., 2017; Sakuraba, 2022). This cost-benefit balance is likely to depend on developmental stage, sink demand, and N status. During early senescence, or under N-sufficient conditions, prolonged chloroplast function may support continued carbon gain. Under N limitation or strong demand from younger or reproductive tissues, however, delayed chloroplast dismantling may retain N in older leaves and delay its remobilization to active sinks (Thomas and Ougham, 2014; Distelfeld et al., 2014).

Evidence for this allocation cost comes mainly from transgenic systems with senescence-inducible expression of the bacterial *ipt* gene. Under growth-limiting N, *P_SAG12_–IPT* tobacco delayed declines in N, protein, Rubisco levels and photosynthesis, but senescing leaves accumulated more ^15^N-labeled N than younger leaves, shoot N accumulation was reduced, and N translocation to non-senescing leaves progressively decreased (Jordi et al., 2000). In wheat, *SAG12::ipt* delayed chlorophyll degradation under limited N and maintained nitrate influx and nitrate reductase activity, but shifted partitioning of newly acquired N toward leaves and away from spikes without improving grain-yield parameters (Sýkorová et al., 2008). This fits the view that delayed senescence can be beneficial when it prolongs source activity, provided that nutrient remobilization to developing sinks is not compromised (Zhou and Yang, 2023). In Arabidopsis, CK effects are currently best documented at the level of chlorophyll retention, photosynthetic-complex stability and photochemical parameters (Oh et al., 2005; Kim et al., 2006; Vylíčilová et al., 2016; Kučerová et al., 2020; Kábrtová et al., 2026), whereas consequences for whole-plant N allocation or reproductive output remain less directly resolved. Nevertheless, older Arabidopsis leaves do remobilize nitrate and other N compounds toward younger N-demanding tissues under low N, and this process is shaped by leaf age, senescence progression and developmental context (Diaz et al., 2008; Fan et al., 2009).

Thus, CK-dependent photosynthetic maintenance and senescence-associated nutrient recycling converge on the same chloroplast protein pool, suggesting that CK availability may influence the timing of the transition between continued source-leaf function and whole-plant N reallocation.

## 4 Chloroplasts as early targets and drivers of senescence

As mature leaves shift from source activity toward nutrient recycling, a transition shaped by developmental age and hormonal regulation, including CKs (Guo et al., 2021; Huang et al., 2022), chloroplasts become both early targets of senescence and active sources of signals that influence its progression (Krieger‐Liszkay et al., 2019; Domínguez and Cejudo, 2021). Chloroplast dismantling proceeds in a progressive and coordinated manner that enables remobilization of N and carbon while limiting cellular damage (Domínguez and Cejudo, 2021). This process involves declining photosynthetic capacity, degradation of chlorophylls, photosynthetic proteins and membrane systems, and activation of chloroplast-derived retrograde, metabolic and redox signals that reshape nuclear gene expression (Janečková et al., 2018; Krieger‐Liszkay et al., 2019; Kučerová et al., 2020; Poudyal et al., 2020; Lei et al., 2023; Tanaka and Ito, 2025; Kábrtová et al., 2026; Yang et al., 2026).

Gradual loss of chlorophyll content is one of the most visible phenomena of leaf senescence. Chlorophyll catabolism proceeds via the well-characterized pheophorbide *a* oxygenase/phyllobilin pathway (Kuai et al., 2018; Tanaka and Ito, 2025), which ensures the safe elimination of phototoxic intermediates.

In detached-leaf dark-induced senescence assays, CKs suppress chlorophyll degradation (Kučerová et al., 2020; Hudeček et al., 2023; Kábrtová et al., 2026), accompanied by reduced expression of selected chlorophyll/LHCII-degradation-associated genes, including *NYE1/2*, *NYC1* and *PAO/ACD1*, depending on the compound and experimental context (Vylíčilová et al., 2016; Hudeček et al., 2023; Kábrtová et al., 2026). Evidence from chloroplast-development contexts further indicates that CKs can support chlorophyll biosynthetic gene expression (Cortleven et al., 2016).

Exogenous CK application can maintain chlorophyll content in detached Arabidopsis leaves for 6–12 days in darkness (Vylíčilová et al., 2016; Nisler et al., 2018; Bryksová et al., 2020; Kučerová et al., 2020; Hudeček et al., 2023; Kábrtová et al., 2026). These assays provide mechanistic evidence for CK-dependent preservation of chloroplast function under controlled conditions, while representing a physiological context distinct from intact age-dependent senescence, as detachment alters sink contact, hormone supply and water or sugar status. Chlorophyll loss precedes major thylakoid reorganization and other ultrastructural changes, and chloroplast dismantling proceeds through both autophagic and non-autophagic routes (Domínguez and Cejudo, 2021; Lei et al., 2023; Izumi et al., 2024).

Photosynthetic activity progressively decreases as senescence proceeds because both carbon-assimilation capacity and primary photosynthetic reactions on thylakoid membranes become impaired. Declining Rubisco activity and content can be an early and major limitation to CO_2_ assimilation during leaf senescence, while Rubisco degradation also contributes to N remobilization from ageing leaves (Friedrich and Huffaker, 1980; Yang et al., 2026). The photosynthetic apparatus is actively remodeled, and individual complexes are degraded with different kinetics. Components of the electron transport chain, including the cytochrome *b_6_f* complex and plastocyanin, can decrease early during senescence (Krieger‐Liszkay et al., 2019), whereas the relative stability of PSI, PSII and light-harvesting complexes (LHCs) varies with species, varieties and environmental conditions (Nath et al., 2013; Krieger‐Liszkay et al., 2019). In detached Arabidopsis leaves, dark-induced senescence is accompanied by an increased chlorophyll a/b ratio, consistent with preferential degradation of LHCII relative to photosynthetic reaction centers (Kučerová et al., 2020; Kábrtová et al., 2026). Perturbation of photosynthetic electron transport also promotes ROS formation, while chloroplast-derived metabolites such as tetrapyrrole intermediates and oxylipins contribute to retrograde signaling, transcriptional reprogramming and nutrient remobilization (Hörtensteiner and Feller, 2002; Bresson et al., 2018; Lin et al., 2024). In this way, chloroplast dysfunction can become self-amplifying, while CK-dependent preservation of chloroplast function can dampen this degradative feedback (Dani et al., 2016; Kučerová et al., 2020; Kábrtová et al., 2026).

## 5 CK-mediated preservation of photosynthetic machinery

CKs counter senescence-associated chloroplast decline at both functional and transcriptional levels, with the magnitude and specificity of these effects influenced by CK structure (Vylíčilová et al., 2016; Bryksová et al., 2020; Kábrtová et al., 2026). It is interesting to note that some N9-protected CKs show improved acropetal transport and improved physiological effects compared to unmodified free bases (Podlešáková et al., 2012; Plíhal et al., 2013). In detached-leaf assays, CK treatments can slow LHC disassembly or maintain LHCs in a competent state depending on compound, treatment regime and light conditions (Oh et al., 2005; Vylíčilová et al., 2016; Bryksová et al., 2020; Kábrtová et al., 2026).

CKs also preserve PSII function at several levels during dark-induced senescence of detached leaves. For example, the CK arabinoside 6-(3-methoxybenzylamino)-9-β-D-arabinofuranosylpurine (3MeOBAPA) sustains excitation supply to the PSII reaction center, reduction of the primary electron acceptor Q_A_ and electron transport beyond Q_A_ in detached Arabidopsis leaves, as monitored by the OJIP transient, although this protection is compound- and species-dependent, with 6-(3-hydroxybenzylamino)-9-β-D-arabinofuranosylpurine (3OHBAPA) being more effective in wheat (Kučerová et al., 2020).

In detached-leaf assays, CK effects have also been reported for the maximum quantum yield of PSII photochemistry (*F_v_/F_m_*) and for partitioning of absorbed light energy into PSII photochemistry (*Φ*_P_), regulated non-photochemical dissipation (*Φ*_NPQ_) and non-regulated non-photochemical dissipation (*Φ*_NO_) (Oh et al., 2005; Lazár, 2015; Vylíčilová et al., 2016; Bryksová et al., 2020; Kučerová et al., 2020; Kábrtová et al., 2026). In contrast, CK effects on Calvin-Benson-cycle maintenance and CO_2_ assimilation in Arabidopsis remain less thoroughly investigated. Therefore, current Arabidopsis evidence supports CK-dependent preservation of pigment content, photosynthetic-complex stability and photochemical competence more strongly than preservation of full carbon-assimilation capacity. Maintenance of CO_2_ fixation during senescence has been documented in other species (Wingler et al., 1998; Jordi et al., 2000; Hudeček et al., 2023; Skowron et al., 2025). In Arabidopsis, transcriptomic and proteomic data show CK-responsive changes in photosynthesis- and chloroplast-related pathways, but direct evidence for preserved CO_2_ assimilation remains limited (Vylíčilová et al., 2016; Kábrtová et al., 2026). The experimental systems and endpoints underlying these distinctions are summarized in **Table 1**.

**Table 1:** Key experimental evidence linking cytokinin action with chloroplast maintenance, photosynthetic performance and nitrogen allocation during leaf senescence.

Study	Species	Genetic system/treatment	Senescence context	Chlorophyll	PSII/PSI/LHC	CO_2_ assimilation/Rubisco	N allocation
Riefler et al., 2006	Arabidopsis	*ahk2*, *ahk3* and *cre1/ahk4* receptor mutants; CK-response assays	Dark-induced senescence in detached leaves	Reduced Chl in *ahk2 ahk3*; CK failed to inhibit dark-induced Chl loss	-	-	-
Kim et al., 2006	Arabidopsis	*AHK3* gain-/loss-of-function lines, *ARR2* overexpression/phospho-defective ARR2 lines; BAP/*t*Z	Age-dependent senescence in plants; dark-induced senescence in detached leaves	AHK3/ARR2-dependent CK signaling delayed Chl loss and leaf senescence	–	–	–
Oh et al., 2005	Arabidopsis	*ore9*; BAP	Dark-induced senescence in detached leaves	BAP delayed Chl degradation in WT and *ore9*	WT: delayed *F_v_/F_m_* decline and stabilized Chl-protein complexes; *ore9*: Chl retained without *F_v_/F_m_* protection	-	-
Vylíčilová et al., 2016	Arabidopsis and wheat	2-chloro-N^6^-(halogenobenzylamino)purine ribosides; BAP reference	Dark-induced senescence in detached leaves	Several derivatives delayed Chl degradation more strongly than BAP	Selected derivatives maintained PSII efficiency and LHCII abundance better than BAP	–	–
Kučerová et al., 2020	Arabidopsis and wheat	CK arabinosides, including 3MeOBAPA and 3OHBAPA	Dark-induced senescence in detached leaves	CK arabinosides delayed Chl degradation	Compound- and species-dependent PSII protection in OJIP analyses	-	-
Kábrtová et al., 2026	Arabidopsis	BAP and MTU	Prolonged darkness and re-illumination in detached leaves	CK treatment delayed Chl loss at later timepoints	Reversible PSII downregulation, enhanced PSI cyclic electron transport and later photochemical competence	–	–
Talla et al., 2016	Rice	*N22-H-dgl162* stay-green mutant; BAP	Dark-induced senescence in detached leaves	BAP delayed Chl loss and maintained Chl-cycle activity	Maintained PSII transcripts, pigment-protein complexes, *F_v_/F_m_* and oxygen evolution	-	-
Wingler et al., 1998	Tobacco	*P_SAG12_-IPT* senescence-induced CK production; iPR leaf-disc assay	Age-dependent senescence in plants; leaf discs light/sugar assays	Age-dependent Chl and protein decline was delayed in *P_SAG12_-IPT*	–	Higher CO_2_ assimilation; delayed decline in Rubisco, HPR and photosynthetic enzymes	–
Jordi et al., 2000	Tobacco	*P_SAG12_-IPT* senescence-induced CK production	Whole plants under limiting N	Chl retention in old leaves; visible senescence delayed	-	Delayed declines in protein, Rubisco and photosynthesis	Older leaves retained more ^15^N; shoot N accumulation and export to young leaves were reduced
Sýkorová et al., 2008	Wheat	*SAG12::ipt* senescence-induced CK production	Whole plants under low N during anthesis/grain filling stage	Chl degradation was delayed	–	–	Nitrate influx and nitrate reductase activity maintained; newly acquired N shifted toward leaves and away from spikes, without yield gain

The PSII/PSI/LHC column summarizes photosystem, light-harvesting-complex and chlorophyll-fluorescence endpoints, whereas the CO_2_ assimilation/Rubisco column summarizes gas-exchange, photosynthetic-rate and Rubisco-related endpoints. The N allocation column includes N remobilization, partitioning and sink allocation. A dash indicates that the endpoint was not a primary focus. Chl, chlorophyll; BAP, 6-benzylaminopurine; *t*Z, *trans*-zeatin; iPR, isopentenyladenosine; MTU, 1-(2-methoxyethyl)-3-(1,2,3-thiadiazol-5-yl)urea; 3MeOBAPA, 6-(3-methoxybenzylamino)-9-β-D-arabinofuranosylpurine; 3OHBAPA, 6-(3-hydroxybenzylamino)-9-β-D-arabinofuranosylpurine; IPT/ipt, isopentenyltransferase; N22, rice cultivar Nagina 22; *N22-H-dgl162*, stay-green mutant in the Nagina 22 background; OJIP, fast chlorophyll a fluorescence induction transient; *F_v_/F_m_*, maximum quantum yield of PSII photochemistry; HPR, hydroxypyruvate reductase.

Recent evidence suggests that CKs can also adjust photosynthetic activity according to light conditions. Both in detached Arabidopsis leaves and intact Arabidopsis plants exposed to prolonged darkness, 6-benzylaminopurine (BAP) and 1-(2-methoxyethyl)-3-(1,2,3-thiadiazol-5-yl)urea (MTU) transiently and reversibly downregulated PSII photochemistry in a phytochrome B (phyB)-dependent manner, while later delaying chlorophyll loss and maintaining functional photochemistry (Kábrtová et al., 2026). This supports placing the response within a phyB-linked light-signaling context, in which the active P_fr_ form of phyB regulates PIF stability and activity (Qiu et al., 2017), while HY5 acts as a positive light-response regulator that can connect light and CK signaling (Cluis et al., 2004; Vandenbussche et al., 2007).

This early decrease in PSII maximum efficiency was associated mainly with a reduction in *F_m_* and increased light-independent energy dissipation, rather than with enhanced regulated non-photochemical quenching, indicating a shift toward constitutive quenching instead of simple loss of PSII reaction-center integrity. In detached Arabidopsis leaves, the reversible decrease in PSII efficiency, together with enhanced cyclic electron transport around PSI, reduced ROS accumulation and subsequent maintenance of photochemical competence, supports the interpretation of a CK-regulated state. In this context, the term “standby-like state” should be understood as a working model for a reversible photochemical adjustment that is accompanied by changes in gene expression, including altered ROS-related transcriptional responses, with *APX1* detected in qPCR analysis and ROS-associated genes such as *ZAT10* and *ZAT12* identified in the transcriptomic dataset, linking photosynthetic adjustment with redox buffering rather than placing ROS control as a separate mechanism (Kábrtová et al., 2026).

At the gene-expression level, CK signaling proceeds through the canonical AHK-AHP-ARR phosphorelay, with AHK2, AHK3 and CRE1/AHK4 activating B-type ARR outputs and A-type ARRs providing negative feedback (Hwang and Sheen, 2001; To et al., 2004; Hutchison et al., 2006; Riefler et al., 2006). Within this pathway, AHK3-dependent phosphorylation of ARR2 directly links CK signaling with chlorophyll retention and extended leaf longevity (Kim et al., 2006). Through this signaling framework, CKs help maintain photosynthetic competence by regulating nuclear genes connected with chloroplast function (Cortleven et al., 2016; Andreeva et al., 2020). One example is the regulation of genes encoding plastid RNA polymerase-associated proteins (PAPs), which are important for assembly and stability of the plastid-encoded RNA polymerase (PEP) complex. Andreeva et al. (2020) showed that CK treatment induced all twelve Arabidopsis *PAP* genes through the canonical CK signaling pathway, although the regulation may be indirect because PAP proteins have different functions and their promoters are complex.

**Figure 1:**
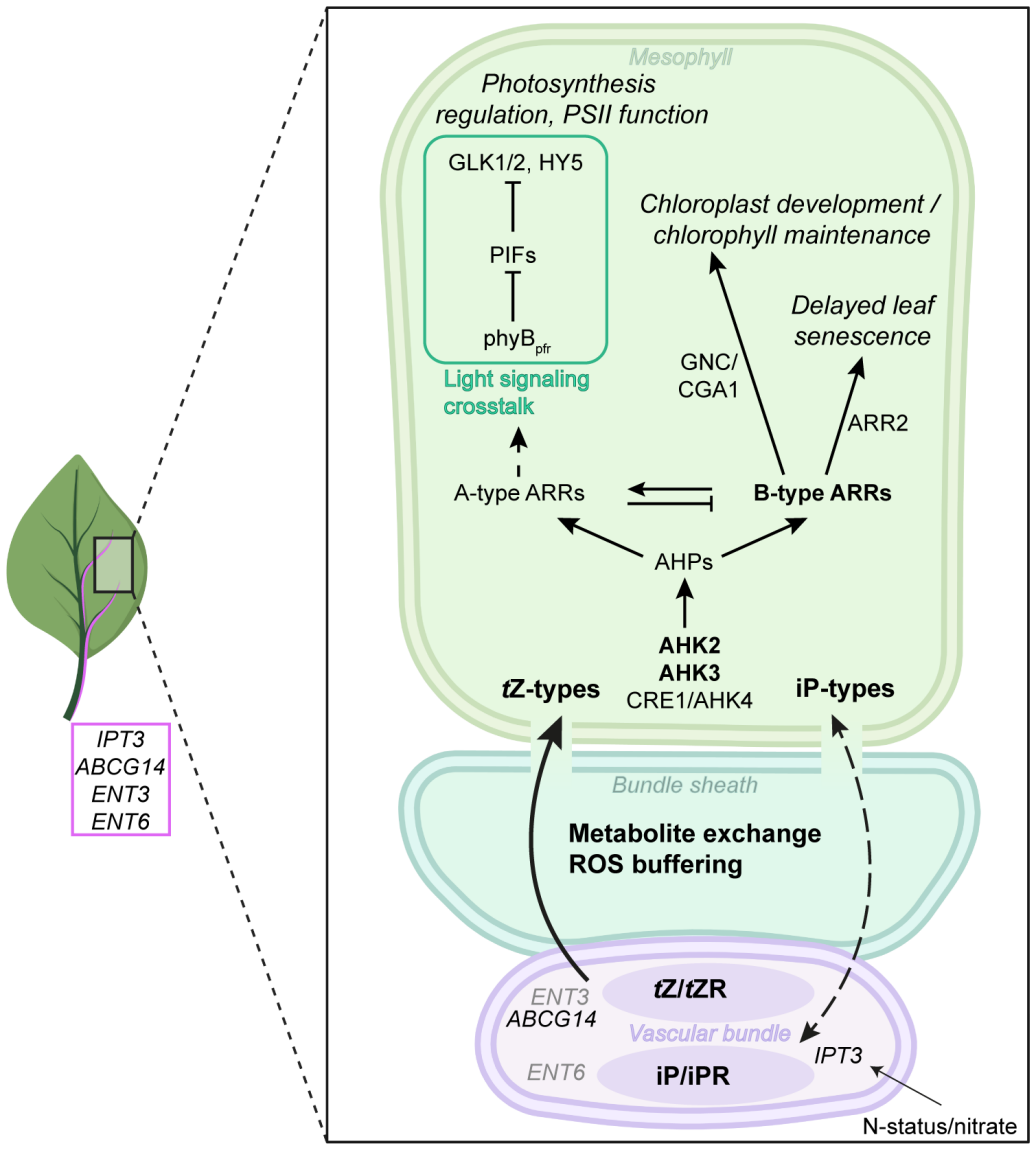
Conceptual scheme of CK transport, signaling and chloroplast-related functions in mature leaves. Nitrate availability promotes cytokinin (CK) biosynthesis, including IPT3-dependent production of shootward CK signals, and supports ABCG14-dependent root-to-shoot translocation of *t*Z-type CKs. In the leaf vasculature, *t*Z/*t*ZR- and iP/iPR-type CK pools may be further redistributed through candidate transport routes, including ABCG14 and members of the Equilibrative Nucleoside Transporter family such as ENT3 and ENT6. In mesophyll cells, CK perception through AHK2, AHK3, and CRE1/AHK4 activates the canonical B-type ARR phosphorelay, while A-type ARRs provide negative feedback regulation. B-type ARR outputs are linked to chloroplast development and chlorophyll maintenance through regulators such as GNC and CGA1, and to delayed leaf senescence mainly through AHK3/ARR2-dependent signaling. CK signaling may also intersect with light-signaling pathways involving phyB and downstream elements, including PIFs, HY5, and GLK1/2, thereby contributing to photosynthetic regulation and PSII function. The scheme integrates evidence from mature and senescing leaves, detached dark-treated leaves, de-etiolation and chloroplast-development studies; line styles indicate evidence strength, with solid arrows denoting experimentally supported links discussed in the text and dashed arrows marking candidate or context-dependent connections. Gray text marks components inferred mainly from expression patterns rather than from directly demonstrated roles in mature or senescing leaves. Created in BioRender. Plíhal, O. (2026) https://BioRender.com/anvx964.

A more direct connection between CK signaling and photosynthesis-related gene expression was shown during de-etiolation, when CKs enhanced light-induced expression of chlorophyll biosynthetic genes, including *HEMA1*, *GUN4*, *GUN5* and *CHLM*, as well as light-harvesting complex genes such as *LHCB1* and *LHCB6* (Chory et al., 1994; Cortleven et al., 2016). This response is mediated mainly through AHK2/AHK3 and the functionally redundant B-type response regulators ARR1, ARR10 and ARR12, with ChIP-qPCR showing binding of ARR10/ARR12 to the *HEMA1* and *LHCB6* promoters (Cortleven et al., 2016). This CK-responsive transcriptional route provides a useful context for the chloroplast-maintenance regulators highlighted in **Figure 1**. Although GLK1/2 and the GATA factors GNC/CGA1 are best established in chloroplast development rather than in CK-delayed senescence, they connect light, nitrate and CK-related signaling with photosynthesis-associated nuclear gene expression, chlorophyll biosynthesis, expression of *GLU1* encoding Fd-GOGAT, and chloroplast growth and division (Waters et al., 2009; Hudson et al., 2011; Chiang et al., 2012).

In mature and senescing leaves, this regulation matters because photosynthetic decline is coupled to chloroplast dismantling and senescence-promoting transcriptional loops. EIN3 and ORE1/NAC2 directly activate major chlorophyll catabolic genes, including *NYE1*, *NYC1* and *PAO*, and ORE1 can further strengthen senescence by activating *NOL* and promoting *ACS2* expression (Qiu et al., 2015). In parallel, the ABA-induced transcription factor AtNAP promotes senescence by activating *CKX3*, which degrades CKs and weakens the CK-mediated anti-senescence signal (Hu et al., 2021). Low-N signaling can also converge on this circuitry. Under N deficiency, APC/C-mediated degradation of GDS1 relieves repression of *PIF4* and *PIF5*, thereby promoting ORE1-dependent early leaf senescence in Arabidopsis (Fan et al., 2023). These pathways suggest that CKs preserve photosynthetic machinery not only by supporting chloroplast-related gene expression, but also by counteracting transcriptional programs that otherwise couple chloroplast dysfunction to accelerated senescence.

## 6 Conclusion and future perspectives

Evidence from Arabidopsis and other systems indicates that CK signaling delays chlorophyll catabolism, slows disassembly of photosynthetic complexes, supports chloroplast-related gene expression, and limits ROS-driven acceleration of senescence. The recent observation in detached Arabidopsis leaves that CKs can transiently and reversibly downregulate PSII photochemistry during prolonged darkness adds an important nuance. In such contexts, CK action may protect leaves by lowering electron pressure and improving redox control before later photosynthetic competence is maintained, indicating that transient photosynthetic down-modulation can be part of the protective response (Krieger‐Liszkay et al., 2019; Domínguez and Cejudo, 2021; Kábrtová et al., 2026).

Several lines of evidence indicate that CKs actively reprogram the transcriptome of mature leaves and buffer redox-dependent feedbacks associated with photosynthetic decline. By limiting excessive ROS accumulation and maintaining chloroplast-related gene expression, CKs may reduce stress pressure on the photosynthetic apparatus and delay the transition toward senescence-associated nutrient remobilization. In this context, CK action may favor maintenance-oriented metabolic states in which photosynthetic competence is preserved before chloroplast dismantling becomes dominant.

Future work should define more precisely how CKs control cellular redox balance and how this regulation shapes functional changes in both photosystems. Further work should determine whether a comparable response occurs during age-dependent senescence in intact plants and whether its reversibility and subsequent recovery after re-illumination distinguish it from persistent photoinhibition or damage. Light quality is particularly important, because CK-dependent effects on PSII photochemistry, PSI-associated electron transport and ROS buffering may depend on the spectral context in which leaves experience darkness, shade, or re-illumination. It will also be necessary to identify which CK forms act locally in ageing leaves, which transporters and cell types control their availability, and how N supply and sink demand modify the balance between photosynthetic maintenance and nutrient remobilization. Ultimately, this should clarify when CKs extend useful source-leaf function and when chloroplast preservation becomes costly for whole-plant resource allocation.
